# A Morphological Approach to the Diagnosis of Protozoal Infections of the Central Nervous System

**DOI:** 10.4061/2011/290853

**Published:** 2011-07-14

**Authors:** Leila Chimelli

**Affiliations:** Department of Pathology, University Hospital, Federal University of Rio de Janeiro, 21941-913 Rio de Janeiro, RJ, Brazil

## Abstract

Protozoal infections, though endemic to certain regions, can be seen all around the world, because of the increase in travel and migration. In addition, immunosuppression associated with various conditions, particularly with HIV infection, favors the occurrence of more severe manifestations and failure to respond to treatments. The CNS may be the only affected system; when not, it is often the most severely affected. Despite information obtained from clinical, laboratory, and imaging procedures that help to narrow the differential diagnosis of intracranial infections, there are cases that need confirmation with biopsy or autopsy. Predominant presentations are meningoencephalitis (trypanosomiasis), encephalopathy (cerebral malaria), or as single or multiple pseudotumoral enhancing lesions (toxoplasmosis, reactivated Chagas' disease). The immune reconstitution disease, resulting from enhancement of pathogen-specific immune responses after HAART, has altered the typical presentation of toxoplasmosis and microsporidiosis. In this paper, a morphological approach for the diagnosis of protozoal infections affecting the CNS (amoebiasis, cerebral malaria, toxoplasmosis, trypanosomiasis, and microsporidiosis) is presented.

## 1. Introduction

Protozoa are single-cell organisms widely distributed in nature. Protozoal infections, though endemic to certain regions for reasons of climate and availability of intermediate hosts to transmit them to man, are also seen outside their original geographical areas, probably facilitated by the increase in international travel and migration of people from their native countries [1–3]. 

These infectious diseases should be largely known, particularly because immunosuppression associated with HIV infection, solid organ, or bone marrow transplant with long-term immunosuppression caused by medications, favor the occurrence of more severe clinical manifestations and failure to respond to specific treatments. A significant proportion of these cases have been described due to immigrants coming from tropical countries to nontropical countries, and increased awareness of these diseases is needed among health professionals dealing with such patients [1, 4, 5]. 

For a number of these organisms, the nervous system is only one of the many systems involved; however, this localization may often be the most severe and incompatible with the survival of the patient [2, 6]. 

Although much information is obtained from clinical examination, laboratory, and imaging procedures [7, 8], all playing a crucial role in identifying and narrowing the differential diagnosis of intracranial infections, there are still cases that need biopsy or autopsy studies to confirm the diagnosis [2, 3, 9]. 

Examination of blood and cerebrospinal fluid (CSF), through direct observation, culture, serology, and molecular diagnosis (including the use of the polymerase chain reaction (PCR)), is extremely important, but tissue samples provide morphological evidence of infection, as well as the substrate for culture and molecular diagnostics. Genetic polymorphisms are increasingly recognized as factors accounting for variation in disease frequency and presentation between individuals [2]. However, a significant number of the diseases are only—and often inevitably so—diagnosed after death. The autopsy brain examination is critical in retrospective diagnosis and audit, so that similar presentations in the future may be more accurately managed. It is important to take into account that disease patterns have changed over time, so that what is presented to neuropathologists for evaluation has shifted dramatically in the last two decades [3, 10]. 

The introduction of highly active antiretroviral therapy (HAART) has improved both the clinical and radiologic findings in HIV-infected patients and reduced the number of opportunistic infections. However, the immune reconstitution inflammatory syndrome (IRIS), or immune reconstitution disease (IRD), the clinical presentation or deterioration of infections resulting from enhancement of pathogen-specific immune responses after the institution of HAART, has altered the typical presentation of some protozoal infections involving the central nervous system (CNS), such as toxoplasmosis and microsporidiosis [4, 11]. 

The predominant presentation of protozoal infections in the CNS may be as meningoencephalitis (e.g., African trypanosomiasis), encephalopathy, such as in cerebral malaria, or as single or multiple space-occupying enhancing lesions in a pseudotumoral form, such as in toxoplasmosis [12, 13]. 

In this paper, a morphological approach for the diagnosis of most protozoal infections that affect the CNS is presented with a brief description of the etiology, epidemiology, and relationship with immunodeficiency. 

A brief taxonomic classification of the major protozoan infections of the CNS is presented in Table 1. 

## 2. Amoebiasis

### 2.1. Cerebral Amoebic Abscess

Cerebral amoebic abscess caused by *Entamoeba histolytica *infection, is a rare global disease, not related to immunodeficiency that causes proctocolitis with bloody dysentery, and liver abscesses and rarely cerebral abscess through haematogenous spread from liver. Transmission is by ingestion of cysts in infected faeces [14, 15]. 

#### 2.1.1. Macroscopic Appearances

The cerebral lesion is usually single and located in the cortical gray matter, basal ganglia, or at the junction between cortex and white matter. Early lesions appear as small foci of hemorrhagic softening that become necrotic, with yellow-green centers, and later cavitate. The walls are irregular and there is no evidence of encapsulation. Occasionally there are multiple abscesses [2, 16]. 

#### 2.1.2. Microscopic Appearances

**Figure 1:**
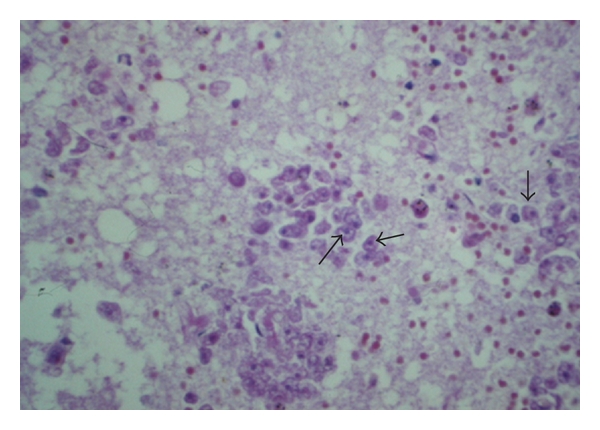
*Entamoeba histolytica* trophozoites are spherical or oval, with granular, sometimes vacuolated cytoplasm and a round single nucleus. The amoebae are PAS positive (arrows).

Cerebral amebic abscesses have an inner zone of necrotic tissue and a broad outer zone with prominent congestion and vascular proliferation. A reactive gliosis and an infiltrate of lymphocytes, plasma cells, macrophages, and some neutrophils are seen in the surrounding brain. Trophozoites can usually be identified in the abscess wall, around vessels, in the necrosis, and at the advancing edge of the lesion. In certain occasions, it may be difficult to distinguish *E. histolytica *trophozoites within this tissue from macrophages. The trophozoites are spherical or oval, 10–60 *μ*m in diameter, with granular eosinophilic, sometimes vacuolated cytoplasm, a round single nucleus with a small central karyosome and peripheral chromatin. Many have phagocytosed erythrocytes and occasionally pseudopodia can be seen. The amoebae are PAS positive, since the cytoplasm contains glycogen (Figure 1). Specific antisera can also be used to identify them by immunocytochemistry [2, 16]. 

### 2.2. Primary Amoebic Encephalitis (Free Living Amoebae)

Infection due to free-living amoebas has increased significantly during the last decades especially in developing countries. Although having in common the ability to run a life independent from the host and the fact that cerebral involvement may be fatal, free living amoebas differ with regard to their epidemiology [17]. 

Acute CNS infection due to Naegleria fowleri, which ends in death within 2–7 days, is termed primary amoebic meningoencephalitis and is not related to immunodeficiency. 

Subacute or chronic CNS infections due to *Acanthamoeba spp, Balamuthia mandrillaris, Leptomixed amoebas, and Sappinia diploidea* are termed granulomatous amebic encephalitis [18, 19], a condition associated with immunodeficiency and also with other chronic debilitating conditions, such as malnutrition and diabetes [20, 21]. 

#### 2.2.1. Primary Amoebic Meningoencephalitis

*Naegleria fowleri *is a free-living amoeba encountered in soil, fresh water, and hot springs. It is found in swimming pools, lakes, and rivers, which are commonly used during the summer months, when the parasite preferentially multiplies. It has a worldwide distribution, occurring as individual cases or small outbreaks. Although the majority of case reports are from the USA, Australia, and Central America this may reflect recognition of the disease. The parasite either exists as a freely motile trophozoite, or in unfavourable circumstances can encyst, hence its ability to survive outside the host. Both the flagellar trophozoite and the cyst forms are infective. The affected individuals are usually young and healthy. The organism invades the nasal mucosa and enters the brain by travelling along the olfactory nerves [17, 21].

**(1) Macroscopic Appearances**

The appearances are those of a fulminant acute meningoencephalitis with a characteristic haemorrhagic necrosis of the olfactory bulbs, tracts, and the adjacent parts of the frontal and temporal lobes. The brain is swollen with a hemorrhagic exudate all over the meninges [21].

**(2) Microscopic Appearances**

**Figure 2:**
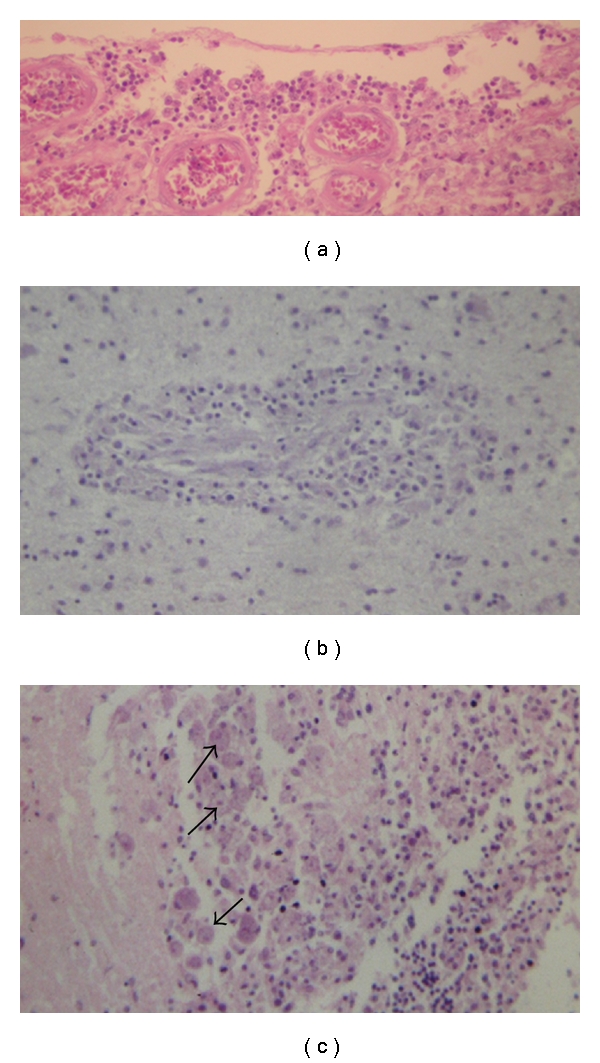
Primary amoebic encephalitis: scant meningeal inflammation (a) extending along the Virchow-Robin spaces (b). *N. fowleri *trophozoites *(arrows) *present within the necrotic tissue resemble macrophages (c). H&E.

Scant inflammation consisting of polymorphonuclear and variable mononuclear infiltrate with focal hemorrhage is seen in the meninges, extending along the Virchow-Robin spaces, associated with haemorrhagic necrosis of the grey and white matter. *N. fowleri *trophozoites measuring 8–15 *μ*m are present in the subarachnoid space and around vessels in the necrotic parenchyma The cytoplasm is vacuolated resembling macrophages, but can be distinguished from them by their vesicular nucleus with its prominent central nucleolus [2, 16] (Figure 2).

#### 2.2.2. Granulomatous Amoebic Encephalitis (GAE)

*Acanthamoeba castellanii, A. polyphaga, *and *Balamuthia mandrillaris, *ubiquitous within the environment in both soil and water, are the most frequent free-living amoebae that cause this pattern of disease. Infection can occur at any time during the year, and mortality due to neurological complications is high. The main risk factors are HIV infection, lymphoma, malnutrition, cirrhosis, and diabetes [2, 21, 22]. Amoebae enter into the lungs via the nasal route, followed by haematogenous spread from where they cross the blood-brain barrier and enter into the CNS. Skin lesions may provide direct entry into the bloodstream, bypassing the lower respiratory tract. The olfactory neuroepithelium may provide an alternative route of entry into the CNS [23].

**(1) Macroscopic Appearances**

**Figure 3:**
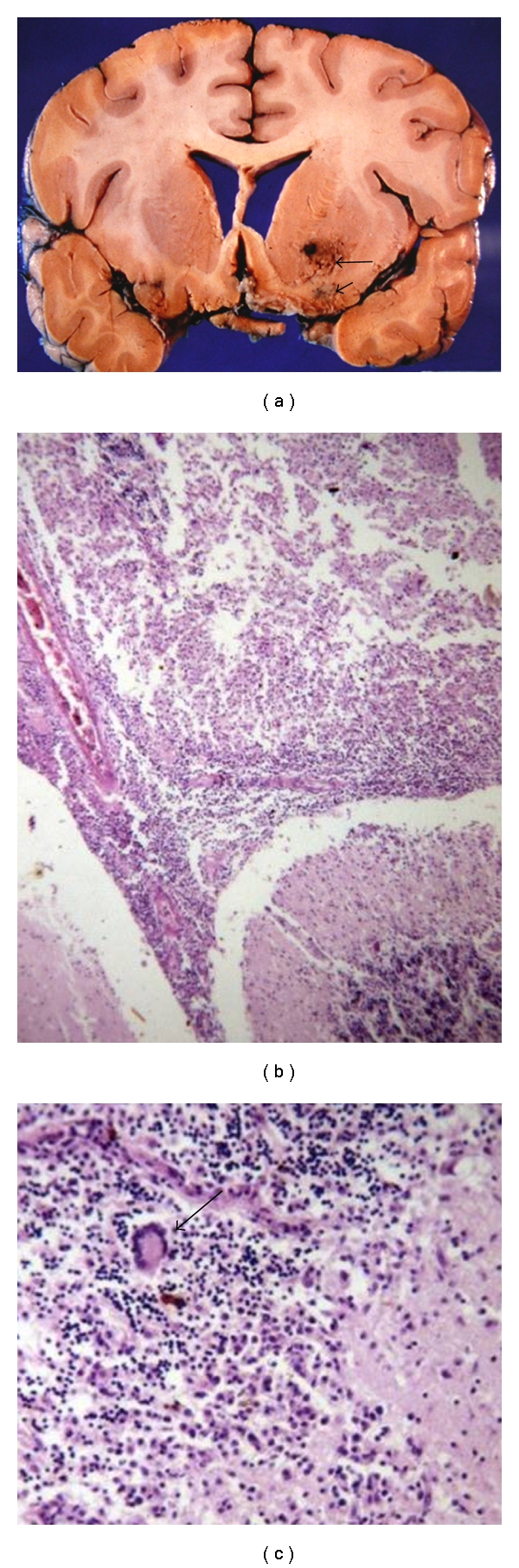
Granulomatous amoebic encephalitis. (a) Foci of haemorrhagic softening (arrows). (b) Necrotic cerebellar folium (b) with a giant cell (c) (arrow), H&E.

The brain is usually swollen, covered by a diffuse leptomeningeal exudate and shows foci of softening, particularly in the anterior part of the cerebral hemispheres, the thalamus, brain stem, and cerebellum. Lesions are haemorrhagic and necrotic (Figure 3(a)).

**(2) Microscopic Appearances**

**Figure 4:**
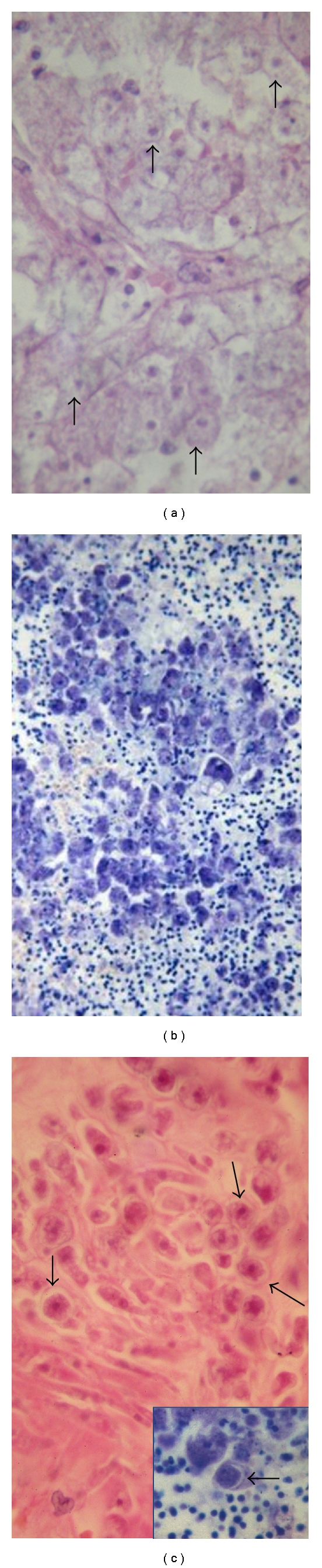
*Acanthamoeba* and *Balamuthia* spp have similar morphology. (a) Trophozoites have a pale granular cytoplasm (arrows), that stain well with Giemsa (b) Cysts have a double thicker cell wall (c), also shown in the insert (arrows). (a) and (c), H&E; (b) and insert, Giemsa.

The meninges and the parenchyma show foci of chronic granulomatous inflammation that tend to be angiocentric and may be necrotizing. The cell reaction is combined acute and chronic, but lymphocytes, macrophages, plasma cells, and foreign body and Langhans-type multinucleated giant cells predominate (Figures 3(b) and 3(c)). *Acanthamoeba *and *Balamuthia *species can encyst. Trophozoites and cysts may be clustered around inflamed vessels that may show fibrinoid necrosis, and also in areas relatively free of inflammation. The trophozoites are 14–40 *μ*m in size that have a pale eosinophilic granular cytoplasm a prominent vesicular nucleus with a dense central nucleolus. Cysts slightly smaller have a double thicker cell wall. Cysts and trophozoites are well demonstrated with the Giemsa and PAS staining (Figure 4). The surrounding brain tissue is necrotic and shows marked astrocytosis and microgliosis. The morphology of the various amoebae that cause GAE is similar. The differentiation can be made by specific immunohistochemistry and molecular diagnostic methods [2, 16, 22].

## 3. Cerebral Malaria

Malaria is caused by infection with the protozoan parasite *Plasmodium *transmitted by the anopheline mosquito. Four species cause human disease: *Plasmodium falciparum, P. vivax, P. malariae, *and *P. ovale*. 

Of these, *P. falciparum* causes the most severe morbidity and mortality and is responsible for the syndrome of cerebral malaria (CM), an important and complex neurological infection that is not related to immunodeficiency. One-third of the world's population is exposed to *P. falciparum *infection, the largest number being in Africa; but South East Asia, India, Central, and South America are also endemic zones [24, 25]. 

It is associated with widespread morbidity and mortality, especially in infants and children, as well as during pregnancy, producing a high morbidity between both mother and fetus. CM is a clinical rather than a pathological diagnosis and should be considered in the differential diagnosis of any patient who has a febrile illness with impaired consciousness who lives in or has recently traveled to malaria endemic areas. 

Morphologically, the common feature is the heterogeneity in neuropathological findings that is in part due to variations in host immunity, the time of death, degree of treatment, and supervening pathology from severe malaria in other organs. Paediatric cerebral malaria differs from those in adults and there is also geographical variation in the clinical and morphological presentation of different populations in Africa and South-East Asia. Genetic differences among the parasites, the degree of host immune response, and underlying genetic polymorphisms, may account for the differences [26]. 

### 3.1. Macroscopic Appearances

**Figure 5:**
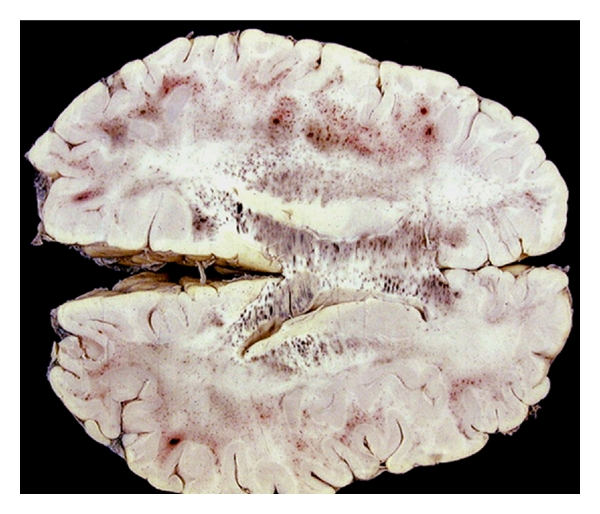
Cerebral malaria: the white matter contains petechial hemorrhages, prominent in the subcortical white matter and *corpus callosum*.

The brain weight may be increased by cerebral swelling, which is variable but usually mild and symmetrical in both adults and children. The meninges are congested and the external colour of the unfixed brain is often a characteristic dusky dark red. In severe cases, petechial haemorrhages are seen on the cortical surfaces. On slicing, there may be obliteration of the sulci and flattening of the gyri. In patients with coexistent severe anaemia, the surface can be pale, whereas in a heavily parasitized brain, the deposition of malaria pigment can give a slate gray color due to the presence of abundant malarial pigment. The cerebral cortex may have an abnormal dusky pink color due to marked congestion. The white matter often contains petechial hemorrhages, prominent in the subcortical white matter, corpus callosum, cerebellum, and brain stem (Figure 5); they are also seen in the cerebellar grey matter in children and are frequent in those whose disease evolves over several days and who have been kept alive in medical care; they are less common and frequently absent in those who have died acutely of cerebral malaria, without medical attention [2, 16]. 

### 3.2. Microscopic Appearances

The central neuropathological feature of CM is the sequestration of parasitized red blood cells (PRBCs) in the microvasculature, which can most easily be recognized under high dry lens or oil immersion, as small ring forms. They are also recognized by the intraerythrocytic pigment (haemozoin) body in the later trophozoite or schizont stages, since erythrocytes infected with the late maturing stages of the parasite disappear from the free circulation, causing a drop in the observed peripheral parasitemia. The specific binding of PRBC to the lining of blood vessels is confirmed with electron microscopy which shows electron dense knob-like protrusions on the surface of infected erythrocytes and at sites of attachment to vascular endothelium [27]. Parasites adhere to specific receptors in the cerebral microvasculature. 

**Figure 6:**
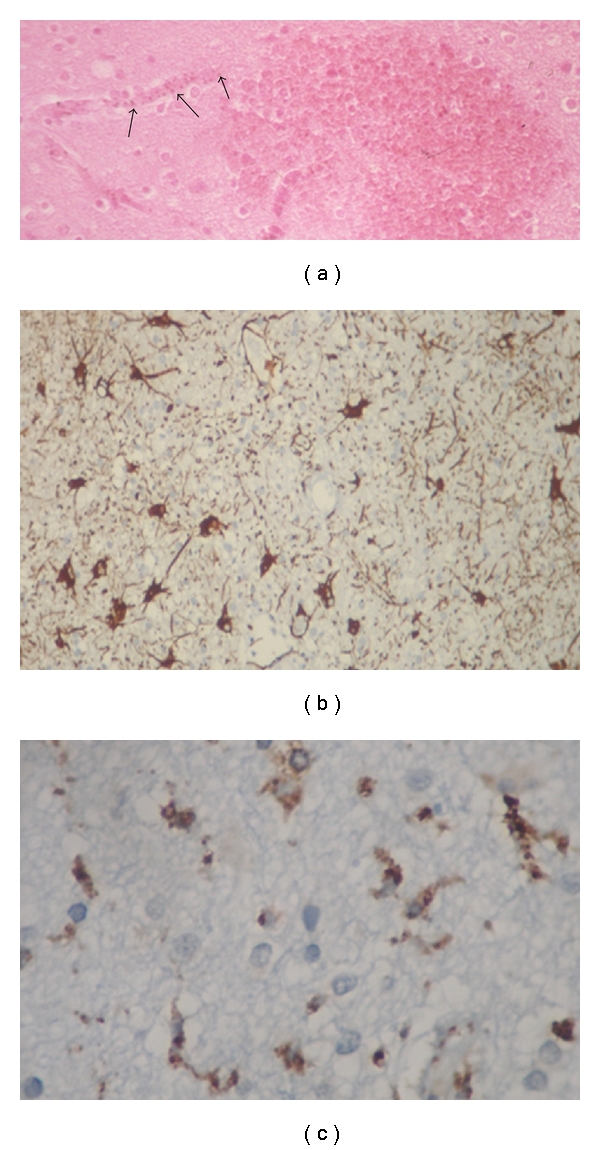
(a) Haemozoin pigment deposition in the lining of the blood vessels (arrows). The pigment may obscure the parasites in the trophozoite stage and can appear similar to formalin pigment, H&E. (b) GFAP and (c) CD68 immunostainings demonstrate astroglial and microglial cell activation, respectively.

Haemozoin pigment deposition occurs microscopically in the lining of the blood vessels (Figure 6), especially in the meninges and choroid plexus. The pigment may obscure the parasites in the trophozoite stage and can appear similar to formalin pigment, although usually forming smaller and darker granules. If there is any doubt over the presence or absence of PRBC, treatment of the slide with picric acid to remove any pigment will then reveal the parasites more clearly. If the brain is examined after 3-4 days of standard therapy, few sequestered PRBC may be seen. In this case, the presence of residual malarial pigment is a diagnostic clue. 

Rupture of infected erythrocytes can lead to an inflammatory process within and around brain capillaries, confirmed with immunocytochemical and molecular biological studies, though cellular inflammation around vessels and in the parenchyma is not a usual feature. If a patient has died after some days of treatment, haemozoin-laden monocytes and neutrophils can be seen in some blood vessels; these cells phagocytose erythrocyte ghosts' adherent to vascular endothelial cells. Patchy endothelial cell activation characterized by swelling, focal endothelial cell damage, and necrosis is sometimes seen, most often associated with incipient areas of haemorrhage. Widespread astroglial activation is seen surrounding blood vessels and microglial nodules may be seen within the brainstem. Markers of microglial cell activation such as CD68, HLA class II antigens, and scavenger receptors are upregulated in the brain in CM. Endothelial cell activation is also evident by the increased expression of intercellular adhesion molecule-1 (ICAM-1). There may be meningeal infiltration by lymphocytes and macrophages [28]. 

**Figure 7:**
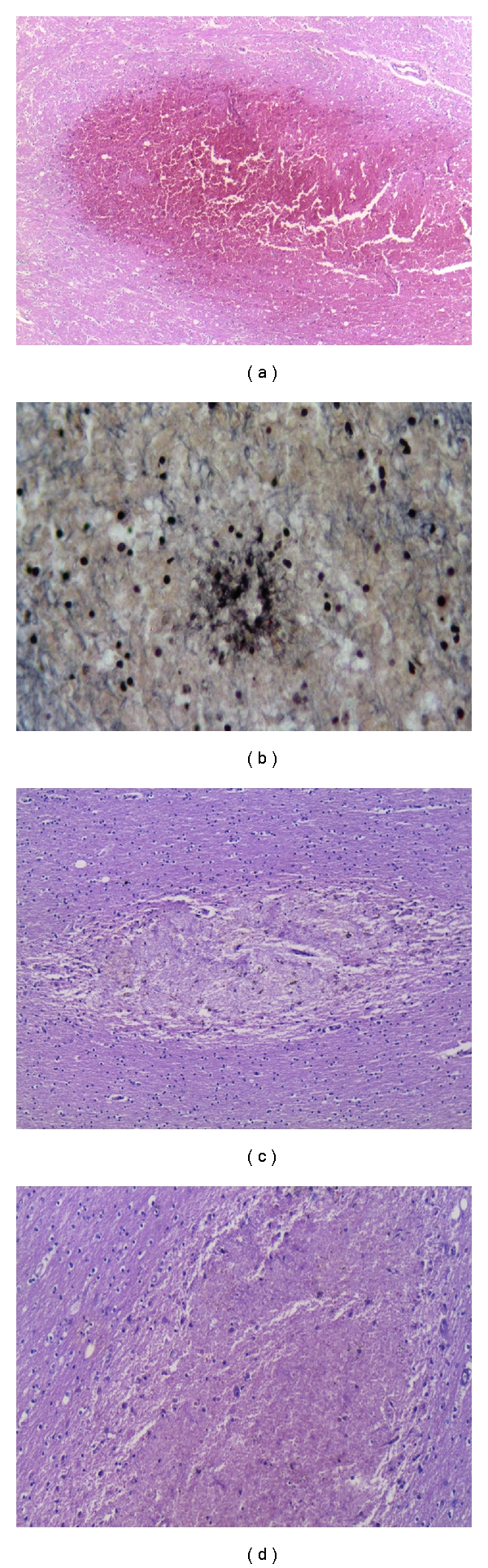
Histology of acute petechiae (a) that may surround necrotic arterioles and venules (b). After resorption (c, d) there may be diffusely scattered cellular reaction after the red cells, infected and uninfected, are cleared from hemorrhage. (a, c, d) (H&E) (b) (PTAH).

Other neuropathological features include petechial hemorrhage in the brain parenchyma, ring hemorrhages, and Dürck's granulomas. Petechial or larger hemorrhages can occur in any part of the brain, but are most common in the white matter and may surround necrotic arterioles and venules (Figure 7). Ring hemorrhage consists of a series of concentric rings surrounding a central necrotic cerebral vessel. The outermost ring contains a mixture of parasitized erythrocytes, free pigment, and host monocytes, with an inner layer of uninfected erythrocytes and gliosis surrounding the vessel. The other lesion peculiar to the brain in malaria is the Dürck's granuloma, which are multiple circumscribed diffusely scattered cellular reaction (collections of astrocytes and microglia containing iron pigment), probably related to resorption of ring hemorrhages. It seems that ring hemorrhages and Dürck's granulomas may represent a temporal spectrum of the same lesion, granulomas being what remains after the red cells, infected and uninfected, are cleared from hemorrhage and this begins to be organized by host response. Similarly, petechial hemorrhages may be the result of vessel rupture in areas of no sequestration, where parasites and their products cannot, or have not yet elicited any host reaction [2, 16]. 

Ischemic changes are not great enough to account for coma as a purely hypoxic event. The reversibility of coma in CM and the large numbers of patients who recover complete neurological function would argue against widespread permanent hypoxic damage, or reperfusion injury, as a pathological mediator in most cases. However, focal necrosis of the brain parenchyma occurs in the white matter. This is ischaemic in origin or follows the petechial haemorrhage and elicits the formation of the Dürck's granuloma. 

Associated axonal damage can sometimes be demonstrated using *β*-amyloid precursor protein staining [29, 30]. 

## 4. Toxoplasmosis

Toxoplasmosis is caused by infection with coccidian parasite *Toxoplasma gondii*. It is distributed globally, with the cat as the definitive host, but any warm-blooded animal, including man, can be an intermediate host. Infection can be acquired in uterus, with severe damage to the developing brain and eye, but most people become infected after childhood and the primary infection is usually clinically silent and latent. Significant disease, of which CNS pathology is the most common and important, happens if the latent infection reactivates when the immune system is compromised for various reasons. Cerebral toxoplasmosis is one of the most frequent opportunistic infections associated with HIV-related immunodeficiency [31]. Presentation as mass lesion is the most common pattern. This requires differentiation from other AIDS-associated mass lesions occurring in the CNS; mainly AIDS-related lymphoma, although progressive multifocal leukoencephalopathy, cerebral tuberculoma and other CNS tumours should also be considered, since cerebral toxoplasmosis can also occur in combination with these other conditions [32].

### 4.1. Macroscopic Appearances

**Figure 8:**
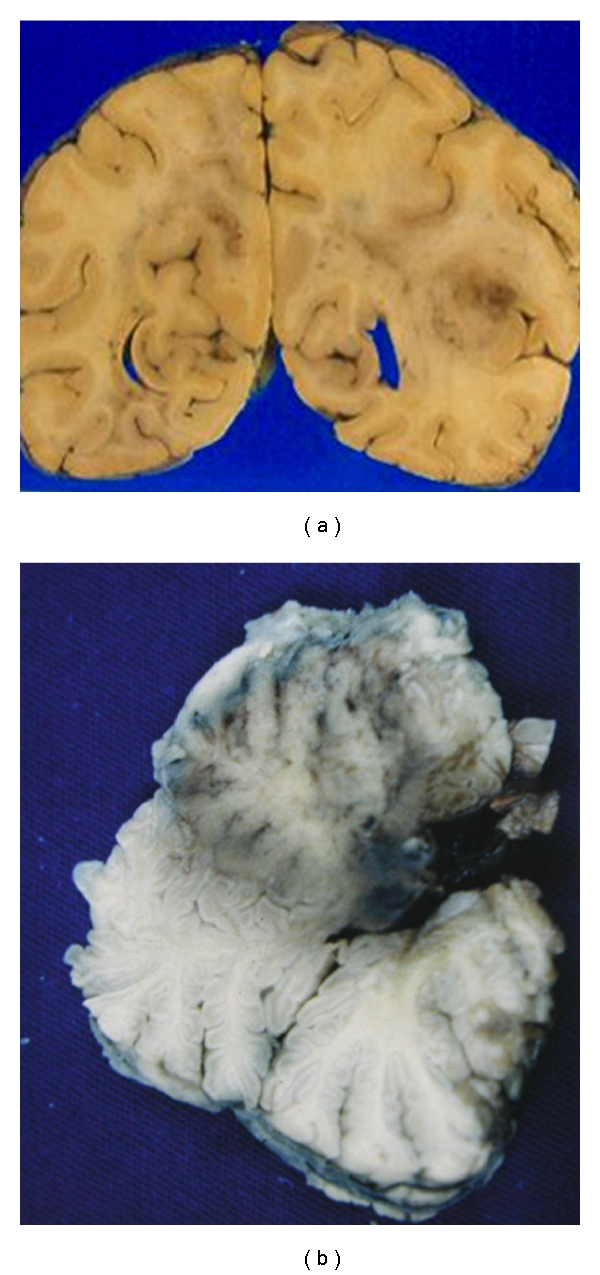
Toxoplasmosis: mass lesions in the right occipital lobe and cerebellar vermis.

**Figure 9:**
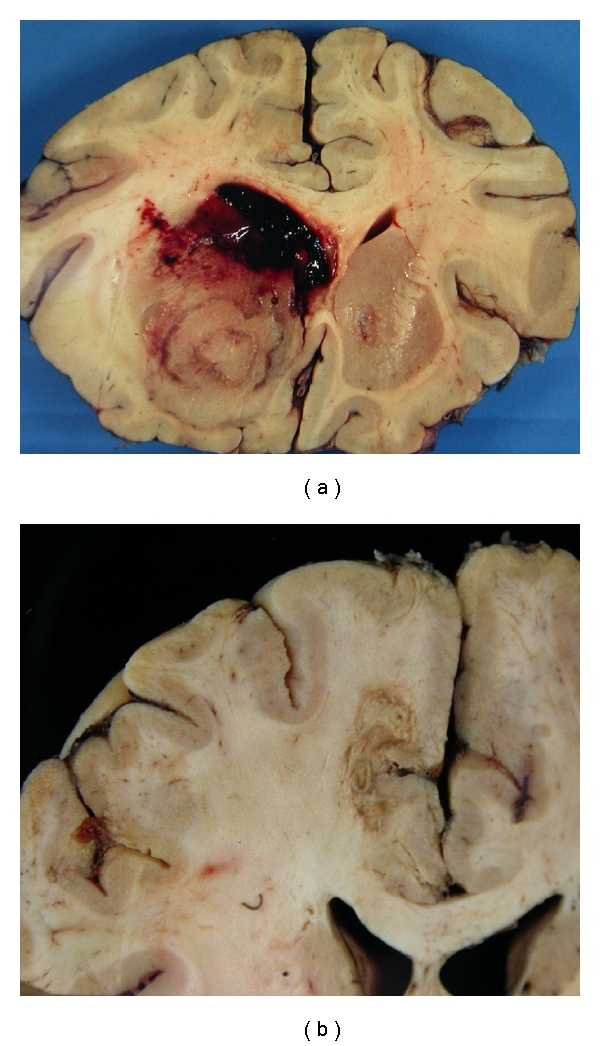
Toxoplasmosis: (a) acute mass lesion in the basal ganglia with associated haemorrhage. (b) Older lesion in process of organization.

The brain contains multifocal single or multiple necrotizing space-occupying lesions of variable sizes. The mass lesions can be located both above and below the tentorium (Figure 8). The basal ganglia and the gray-white matter cortical junctions are often involved, but any part of the brain may be affected. There may be associated hemorrhage. Older lesions are cystic due to resorption of necrotic material (Figure 9). Occasionally brain involvement results in an encephalitic process without obvious focal lesions on macroscopic examination [2, 16]. 

### 4.2. Microscopic Appearances

**Figure 10:**
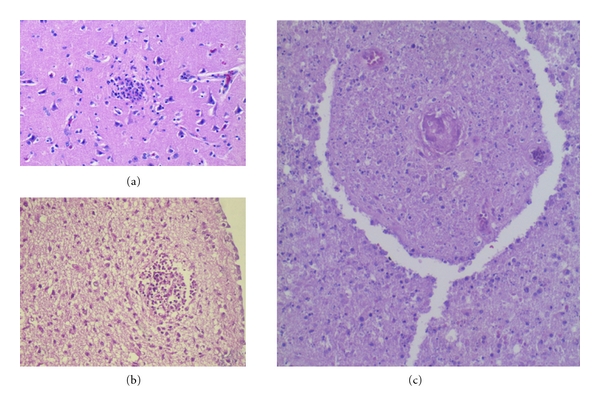
Toxoplasmosis—Microglial nodules (a, b) and “dirty” coagulative necrosis (c) containing a necrotic vessel with the features of endarteritis *obliterans* and thrombosis. H&E.

There is much heterogeneity of CNS toxoplasma lesions, with overlapping patterns and sometimes temporal heterogeneity. The basic process is cell infection and associated inflammation, forming microglial nodules with surrounding astrocytosis. Necrosis of infected cells and surrounding tissues is usual, leading to expansion of the necrotic foci into the mass lesions that are usually seen. The necrosis is typically coagulative and “dirty”, with abundant fragments of nuclear debris (some of which are actually toxoplasma tachyzoites). Around the necrosis there are mononuclear and polymorphonuclear inflammatory cells, newly formed capillaries, edema, reactive astrocytes, and microglia. Vessels are surrounded or infiltrated by lymphocytes and macrophages with the appearances of a vasculitis, occasionally with fibrinoid necrosis, intimal proliferation, and thrombosis, with the features of endarteritis obliterans. Affected vessel may rupture, causing perivascular or larger haemorrhage (Figure 10). 

**Figure 11:**
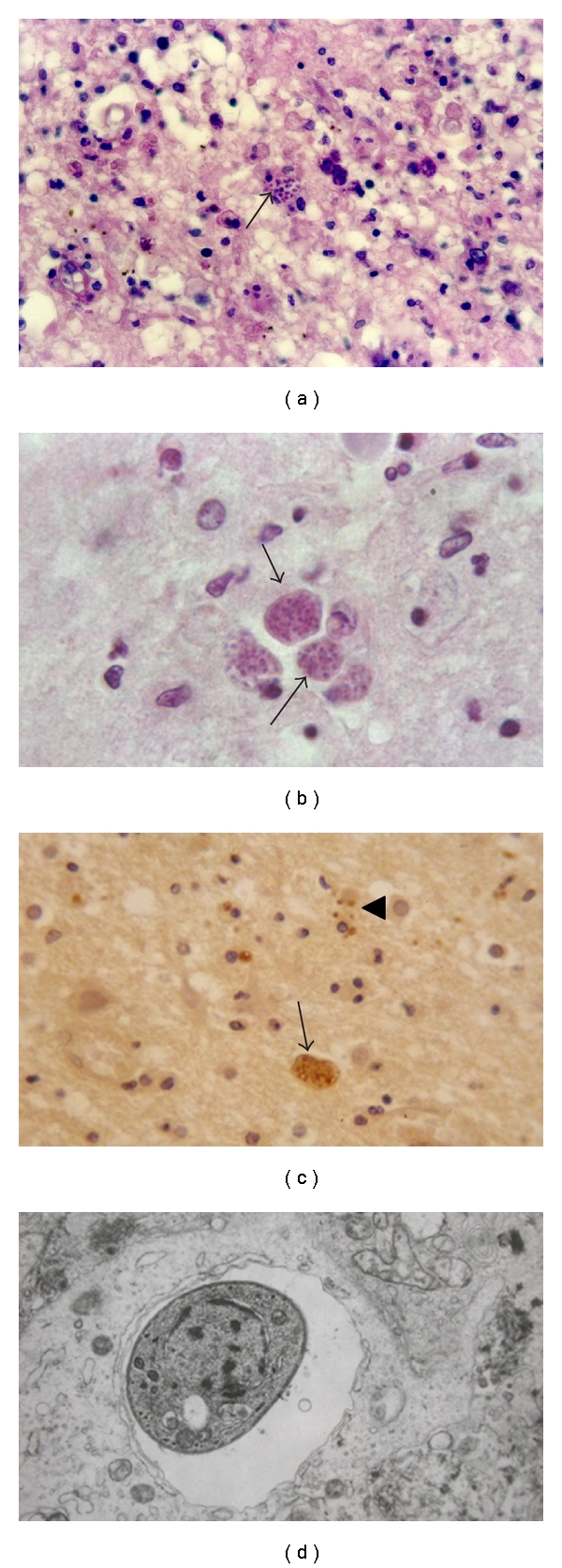
(a) Extracellular Toxoplasma tachyzoites (arrow) in the inflamed tissue around the necrosis. (b) Pseudocysts (arrows); (c) immunocytochemical staining with anti-*T. gondii* antibodies identifying tachyzoites (arrowhead) and pseudocysts (arrow). (d) At ultrastructural level a parasite within a vacuole in the cell cytoplasm (a, b), H&E.

Intracellular and extracellular Toxoplasma tachyzoites (also known as endozoites or trophozoites) and pseudocysts (containing large numbers of bradyzoites, also known as cystozoites), may be easily found with hematoxylin and eosin staining, in the inflamed tissue around the necrosis. Their frequency varies, sometimes abundant, particularly pseudo-cysts, but on occasions are scanty as in treated lesions, when immunocytochemical staining with anti-*T. gondii *antibodies is useful in identifying parasites. Tachyzoites are oval- or crescent-shaped and measure 2–4 *μ*m. Those within cells may be clustered together (in vacuoles or larger pseudocysts measuring 20–100 *μ*m in diameter) or may appear to lie free in the cell cytoplasm (Figure 11). 

**Figure 12:**
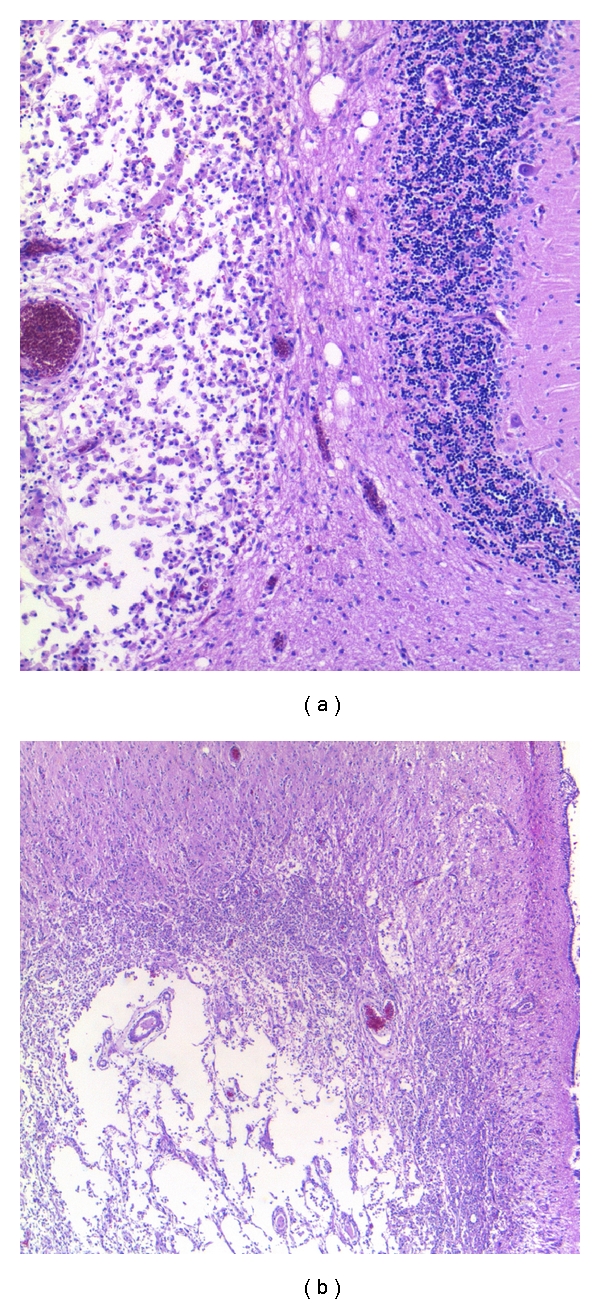
Toxoplasmosis. Chronic, treated, cystic lesions containing macrophages in the cerebellum (a) and periventricular regions (b).

In chronic, treated lesions, the central area of coagulative necrosis is well demarcated, may become cystic, containing macrophages (Figure 12), and surrounded by microglial nodules and reactive astrocytes. Organisms are scanty or absent and immunostaining may or may not identify residual antigen in these lesions. Healed or end-stage (burnt out) lesions, when tissue reaction is no longer available, are common presentation nowadays, particularly in countries where the treatment is effective. With the antiretroviral therapy some lesions are associated with the IRIS. 

Cerebral toxoplasmosis occasionally causes a diffuse nonnecrotizing encephalitic pattern with scattered microglial nodules that include parasites and astrocytosis involving both gray and white matter. In a less frequent peri-ventricular pattern, there is a rim of necrosis up to 1 cm thick along the lateral and third ventricles, where abundant parasites are visible [2, 16]. 

### 4.3. Congenital Toxoplasmosis

It is rare (less than 5 cases per 100,000 live births), occurs only if maternal infection is acquired during pregnancy, and is transmitted through the placenta [33]. The risk of maternal exposure is geographically variable and may occur at any time during pregnancy, although the highest risk is after the first trimester [34, 35], rising to about 30% and late in the third trimester it approaches 100%. However, a fetal *Toxoplasma *infection early in pregnancy is likely to cause major disruption of CNS organogenesis with resulting fetal death or hydrops and severe cerebral abnormalities. Parasites proliferate and spread widely in the absence of circulating antibody. The severity of complications declines with advancing pregnancy, although premature delivery, chorioretinitis, minor brain calcifications, and even fetal death may still result from late infection [33, 36].

#### 4.3.1. Macroscopic Appearances

Macroscopic abnormalities occur in the more severe cases and include multifocal or confluent areas of necrosis, particularly in the periventricular and subpial regions. The brain may be collapsed because of total parenchymal destruction secondary to vascular thrombosis. Periventricular and periaqueductal ulceration, hydrocephalus, or even hydranencephaly may occur. Microcephaly is found in cases with severe brain destruction. Foci of calcification may be scattered throughout the brain in contrast to the predominantly periventricular calcification of congenital CMV [2, 16]. 

#### 4.3.2. Microscopic Appearances

Necrotic areas are usually associated with lipid-laden macrophages, lymphocytes, and a few neutrophils, which are also present in the leptomeninges. Contact with circulating antibody passively transferred from the mother may account for perivascular inflammation and thrombosis. The adjacent brain tissue may contain microglial nodules. Toxoplasma tachyzoites and cysts can be seen in the meningeal exudate, around the necrotic lesions, and are particularly numerous near the ventricular cavities. They are hard to detect within frankly necrotic areas. Antitoxoplasma antibodies detect both free (tachyzoite) and encysted (bradyzoite) organisms. The foci of necrosis eventually tend to undergo mineralization, although residual toxoplasma cysts can still be found. Ependymal granulations and gliosis may lead to aqueduct stenosis and obstructive hydrocephalus [2, 16].

## 5. Trypanosomiasis

Trypanosomes are hemoflagellates, that causes disease in large, but geographically restricted, parts of the world. There are three species of *Trypanosoma (T. brucei gambiense and rhodesiense and T. cruzi), *that affect man, all transmitted by blood-feeding insects. Though morphologically similar in their trypomastigote blood form, they give rise to quite different diseases in Africa and South America, respectively [37, 38]. 

### 5.1. Human African Trypanosomiasis (Sleeping Sickness)

Human African trypanosomiasis (HAT) is caused by two blood parasites *Trypanosoma brucei rhodesiense *and *Trypanosoma brucei gambiense*. Morphologically identical but causing significantly different clinical syndromes, they are found in west Africa (*T. b. gambiense*) and in eastern and southern Africa (*T. b. rhodesiense*) [39]. *T. b. rhodesiense *is a zoonosis, cattle being the reservoir (with significant veterinary consequences). 

HAT is transmitted from man to man or cattle to man by *Glossina *spp tsetse flies. It is estimated that millions of people are at risk of infection, including tourists visiting game parks in East and Central Africa. Both infections cause a systemic and meningoencephalitic syndrome in man in late phases of the disease, with high mortality when untreated. Involvement of the central nervous system (CNS) usually follows 3-4 weeks after infection by *T. b. rhodesiense*, whereas it takes many months or years in case of *T. b. gambiense*. The CNS manifestations are protean, nonfocal, and easily misdiagnosed when the patient is encountered out of context, as in migrants to nonendemic countries. The early acute disorder, usually more severe in case of *T. b. rhodesiense *infection, has little or no impact on the CNS, while infection by *T. b. gambiense *is responsible for subacute or chronic meningoencephalitis [40, 41]. 

#### 5.1.1. Macroscopic Appearances

There are few postmortem reports examining CNS material from human cases available. Macroscopic changes may be scanty or absent. The leptomeninges are congested and may be cloudy, with slightly opaque fluid, more evident at the base and over the cerebellum. The brain is swollen and congested. No obvious abnormalities are seen on brain section. 

Patients who have been treated with melarsoprol may develop acute hemorrhagic leukoencephalopathy, which occurs in 5–10% of late stage treated patients. It is suggested that this reactive lethal encephalopathy is triggered by the viable organisms remaining within the brain after administration of insufficient doses of drugs [16, 42]. 

#### 5.1.2. Microscopic Appearances

Histological features are those of a diffuse meningoencephalitis consisting of lymphocytes, including large numbers of B cells, plasma cells, and histiocytes surrounding blood vessels and infiltrating the subarachnoid spaces. The vessel walls are inflamed but not necrotic. Lymphocytes and plasma cells are often spread out from the vessels into the adjacent grey and white matter and there is diffuse microglial hyperplasia, proliferation of large astrocytes, and formation of microglial nodules. A characteristic, but by no means pathognomonic, feature of trypanosomiasis is the morular (or Mott) cell, a modified plasma cell with a small, peripheral nucleus, and cytoplasm filled with globules of immunoglobulin (Russell bodies). These cells are present in the inflammatory infiltrate in the meninges and brain, but may also be found isolated in otherwise unaffected areas, especially in *T. b. rhodesiense *encephalitis, often associated with lymphophagocytosis. The diffuse reactive astrocytosis, is known to be a common event in this disease; once activated, astrocytes may express and secrete a number of cytokines. Conversely, a number of cytokines induce gliosis. Within the CNS trypanosoma is responsible for involving virtually all its components; injury to the choroid plexuses will enable the parasite to invade the CSF at a very early stage of the infection; parasites may reach the brain parenchyma through the Virchow-Robin spaces and this may represent the progression from meningitis to encephalitis. Parasite products can activate CD8+ T-cells, which secrete interferon-*γ* and interleukin-2 [43]; these activate macrophages to produce nitric oxide, tumour necrosis factor-*α*, and other products, which stimulate the astrogliotic reaction. Trypanosomes are rarely demonstrable in the histological sections and are only rarely seen in the meninges. Apparently, the differences between the encephalitis produced by the two organisms are exclusively quantitative and probably related to the stage at which the observations were made [2, 16]. 

### 5.2. American Trypanosomiasis (Chagas' Disease)

Chagas' disease is caused by the protozoan *Trypanosoma cruzi*, which can take up the undulating, blood stream flagellate trypomastigote form, or the *Leishmania*-like amastigote form. Chagas' disease or American trypanosomiasis is widespread throughout Latin America, particularly in rural areas, but international migration is exporting the disease to developed countries [5, 38]. Night-biting triatomine bugs living on livestock or in cracks in walls are the vectors. Mature parasites are excreted in the faeces of the bugs whilst they feed, and inoculated directly through broken skin because of itching and scratching, or via transfer on fingers into the conjunctival sac. 

Acute infection tends to be a mild self-limiting febrile illness. Approximately 30% of infected individuals develop chronic Chagas disease, which most commonly affects the heart (causing cardiomyopathy and dysrhythmias) or the digestive system (causing mega-oesophagus or mega-colon). Immune suppression from any cause may result in reactivation of latent infection, causing extensive necrotizing encephalitis [38, 44, 45]. 

#### 5.2.1. Neuropathology of CNS Lesions

CNS involvement may occur in the acute infective stage, not so clear in the chronic stage, and in reactivation of latent infection in the chronic phase in immunosuppressed patients. In the last context, new pathological presentations of the disease have been recorded in HIV-infected patients [45].

**(1) Macroscopic Appearances**

The changes are not conspicuous, except in the reactivated form, which will be described below. In the acute phase the brain appears swollen and congested, with scattered petechial hemorrhages. In the chronic phase there are usually no macroscopic changes.

**(2) Microscopic Appearances**

In the acute phase, there is a diffuse meningoencephalitis with multiple inflammatory foci, lymphocytes, some polymorphs, and macrophages throughout the CNS, with the tissue damage apparently due directly to destruction caused by the parasite after rupture of the cells. There are perivascular infiltrates of lymphocytes scattered within the brain parenchyma and amastigote (Leishmania-like) forms of the parasites within astroglial cells or less frequently at the center of microglial nodules, which are also scattered within the brain parenchyma, macrophages and endothelial cells. Lesions can also be seen in the meninges, and choroid plexus. Amastigote forms can be detected by conventional histology, by immunofluorescence and *in situ *hybridization.

The existence of a chronic form affecting the CNS including a range of unexplained clinical presentations has not been well documented morphologically. Few small, hypocellular microglial nodules and aggregates of lymphoid cells sparsely distributed in the nervous tissue of some patients may be found, but no parasites, except in one case [46]. Neuronal loss is observed, but may be due to chronic hypoxia secondary to the cardiomyopathy accompanying chronic Chagas' disease. These relatively insignificant inflammatory changes are interpreted as being of a residual nature, possibly representing sequelae of the inflammatory nodules of the acute form, reinforcing the view against the existence of an anatomical basis for a chronic nervous form in Chagas' disease [2, 44, 45].

**(3) Reactivated Disease**

It occurs in patients chronically infected with *T. cruzi *who are immunosuppressed because of malignant neoplasms of the hematopoietic-lymphoid system, renal, heart, and bone marrow transplantation and especially because of HIV infection. It should be considered as a differential diagnosis of meningoencephalitis and space-occupying lesions in HIV patients with low CD4 T-cell counts, living in or coming from an endemic area, especially if a mass lesion does not respond to therapy against toxoplasmosis [47–50].

**Figure 13:**
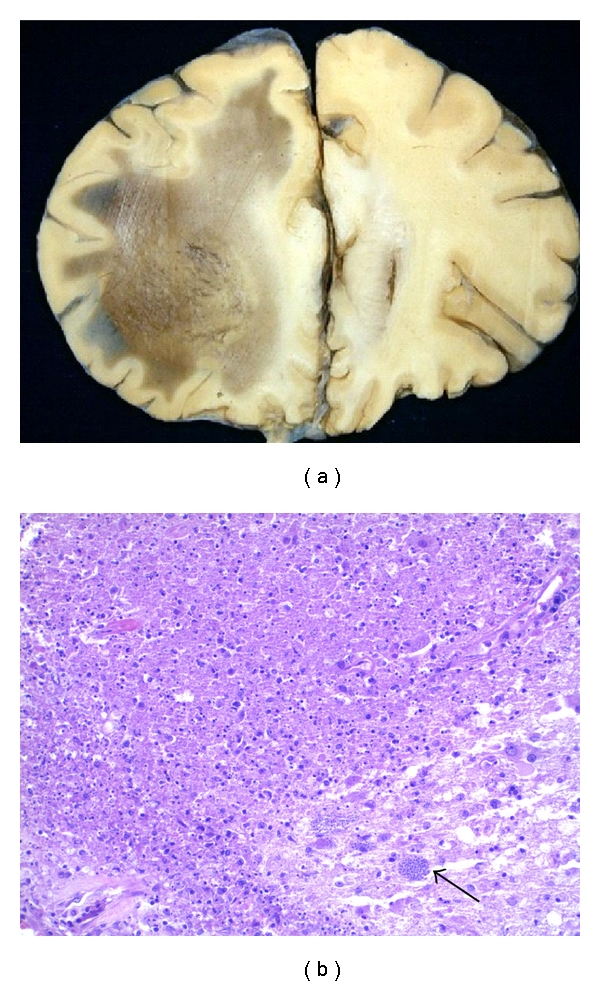
Reactivated Chagas' disease. (a) An extensive acute necrotizing encephalitis seen histologically in (b), where amastigote forms of* T. cruzi* (arrow) are present, H&E.

Macroscopically, it takes the form of extensive necrotizing encephalitis and many patients have the pseudotumoral form, characterized by the presence of single or multiple necrotic-hemorrhagic nodular lesions, mostly located in the cerebral lobes and, in some cases, in the brain stem and cerebellum. The lesions are poorly limited, measuring several centimeters, and preferentially involve the white matter (Figure 13). 

**Figure 14:**
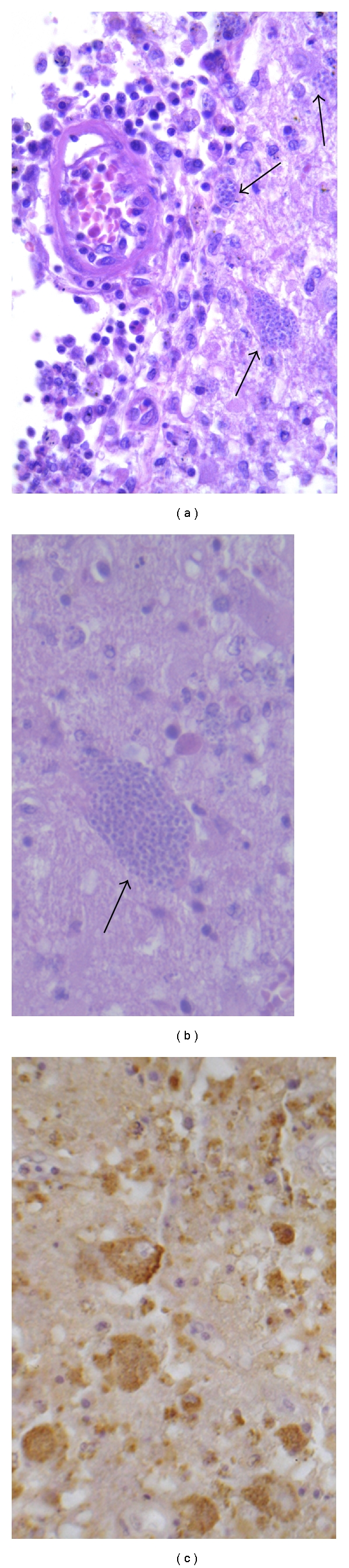
Reactivated Chagas'disease. Lymphoplasmacytic leptomeningitis with subpial amastigote forms of the parasite (a, b) (arrows). The identity of the parasite was confirmed immunohistochemically (c) (a, b), H&E.

Histologically, this is an acute necrohaemorrhagic encephalitis characterized by microglial nodules, with associated haemorrhage, necrosis, and exudates of macrophages, lymphocytes, plasma cells and, to a lesser extent, neutrophilic granulocytes within the nervous tissue and perivascular spaces. Amastigote forms of the parasite are abundant within macrophages and astroglia but are not found in nerve cells. The identity of the parasite can be confirmed immunohistochemically. Lymphoplasmacytic leptomeningitis is a constant finding and apparently represents an extension of the subjacent necrotic-inflammatory lesions [2, 16, 45] (Figure 14).

**(4) Other Causes of CNS Pathology**

In chronic Chagas' disease, they include vascular associated lesions (ischemic or hemorrhagic) that are related to the chronic myocardiopathy that courses with congestive heart failure, dysrhythmias, and thromboembolic phenomena. These include secondary hypoxic nerve cell changes, cerebral infarcts, and hemorrhages [2, 45, 51, 52].

In addition, mild meningoencephalitis with epithelioid and giant cells may occur in cases of congenital Chagas' disease [53, 54]. The risk of infected mothers in the chronic phase, transmitting the disease to their fetus during pregnancy is however very low, probably <1% [55]. 

Components of the *peripheral nervous system* may also be affected in the acute phase of Chagas' disease. The autonomic tissue of the heart, oesophagus, and gut are especially susceptible, leading to the chronic cardiopathy with cardiomegaly, and the digestive megaviscera. The peripheral somatosensory neuropathies, including involvement of dorsal root ganglia, anterior horn neurons and peripheral sensory and motor nerve fibres are less frequent than the autonomic, but have been well documented clinically and electrophysiologically in chronic cases, supported by morphological reports in humans and experimental infection, showing both axonal and demyelinating neuropathies. They are postulated to result from several autoimmune mechanisms [2].

## 6. Microsporidiosis

Microsporidia are single-celled, obligate intracellular parasites. More than 20 genera of microsporidium are pathogenic in mammals, but *Encephalitozoon *species affect immunosuppressed populations more commonly than other species [1, 56]. *Trachipleistophora anthropophthera *may also cause encephalitis. In humans, microsporidium can be transmitted via contaminated water or air droplets and via the fecal-oral route. Sexual transmission of *Encephalitozoon *species may also occur. 

The parasites have an internal coiled tube through which sporoplasm is extruded to pierce and inject infectious sporoplasm into the cytoplasm of the host cell. Disseminated infection with CNS involvement has been reported following kidney, pancreas, and bone marrow transplantation. Rare cases of CNS involvement have been reported in immunocompetent hosts [2]. 

### 6.1. Macroscopic Appearances

Microsporidiosis presents in the CNS as diffuse, nodular encephalitis, sometimes with necrotic foci. Multifocal lesions in gray or white matter can mimic cerebral toxoplasmosis. 

Mild meningeal opacity has been reported. 

### 6.2. Microscopic Appearances

There may be small foci of necrosis. The parasites may be detected in tissue biopsy specimens. Astrocytes are parasitized (but not neurons) and there is localized microglial proliferation. They are seen in clusters as haematoxyphilic nuclear dots within refractive clear cytoplasm, often Gram- and Ziehl-Neelsen-positive. Electron microscopy is more successful than light microscopy to identify the organisms [56]; molecular diagnosis with PCR of tissue biopsy samples is highly sensitive but is not widely available. During CNS infection, spores are often present in peripheral blood. 

## 7. Leishmaniasis

While visceral leishmaniasis (*Leishmania donovani*) is a relatively common infection in all continents except Australasia, and spreads haematogenously in the body, CNS involvement is extremely rare [1]. 

Experimentally, *L. amazonensis *can cause encephalitis with parasites in the cerebral parenchyma [57] but there is only one recorded case of CNS infection by *L. donovani *in man. A child with drug-refractory visceral leishmaniasis had meningitis associated with parasites in the CSF. 

## Figures and Tables

**Table 1:** Protozoal infections that may affect the central nervous system.

Amoebiasis Cerebral abscess * Entamoeba histolytica * Primary amoebic encephalitis (free living *amoebae*) (a) Primary amoebic meningoencephalitis * Naegleria fowleri * (b) Granulomatous amoebic encephalitis * Acanthamoeba *spp. * Balamuthia mandrillaris * * Leptomixed amoebas * * Sappinia diploidea *	
Cerebral malaria * Plasmodium falciparum *	
Toxoplasmosis * Toxoplasma gondii *	
Trypanosomiasis African trypanosomiasis (Sleeping sickness) * Trypanosoma brucei gambiense * * Trypanosoma brucei rhodesiense * South American trypanosomiasis (Chagas disease) *Trypanosoma cruzi. *	
Microsporidiosis * Encephalitozoon spp *	
Leishmaniasis * Leishmania spp *	

Note that cerebral leishmaniasis is exceedingly rare, and microsporidial infections have become apparent as significant human CNS diseases only in the last decades.
